# Brain training with non-action video games enhances aspects of cognition in older adults: a randomized controlled trial

**DOI:** 10.3389/fnagi.2014.00277

**Published:** 2014-10-14

**Authors:** Soledad Ballesteros, Antonio Prieto, Julia Mayas, Pilar Toril, Carmen Pita, Laura Ponce de León, José M. Reales, John Waterworth

**Affiliations:** ^1^Studies on Aging and Neurodegenerative Diseases Research Group, Department of Basic Psychology II, Universidad Nacional de Educación a DistanciaMadrid, Spain; ^2^Department of Informatics, Umea UniversityUmea, Sweden

**Keywords:** attention, brain plasticity, cognitive aging, non-action video games, speed of processing, training, wellbeing

## Abstract

Age-related cognitive and brain declines can result in functional deterioration in many cognitive domains, dependency, and dementia. A major goal of aging research is to investigate methods that help to maintain brain health, cognition, independent living and wellbeing in older adults. This randomized controlled study investigated the effects of 20 1-h non-action video game training sessions with games selected from a commercially available package (*Lumosity*) on a series of age-declined cognitive functions and subjective wellbeing. Two groups of healthy older adults participated in the study, the experimental group who received the training and the control group who attended three meetings with the research team along the study. Groups were similar at baseline on demographics, vocabulary, global cognition, and depression status. All participants were assessed individually before and after the intervention, or a similar period of time, using neuropsychological tests and laboratory tasks to investigate possible transfer effects. The results showed significant improvements in the trained group, and no variation in the control group, in processing speed (choice reaction time), attention (reduction of distraction and increase of alertness), immediate and delayed visual recognition memory, as well as a trend to improve in *Affection* and *Assertivity*, two dimensions of the Wellbeing Scale. Visuospatial working memory (WM) and executive control (shifting strategy) did not improve. Overall, the current results support the idea that training healthy older adults with non-action video games will enhance some cognitive abilities but not others.

## Introduction

The current computer-based randomized controlled trial was conducted to determine the effects of training with non-action video games on cognitive functioning in healthy older adults. To evaluate possible transfer effects, we assessed a broad variety of cognitive functions that decline with age as well as subjective wellbeing in a pretest-training-post-test randomized controlled trial design with an experimental group and a control group.

Normal aging is associated with age-related gray and white matter shrinkage, with the prefrontal cortex showing greater change than posterior regions. The lateral prefrontal cortex, the cerebellum and the medial temporal lobe system, including the hippocampus, are also affected while minimal or no reduction of volume occurs in entorhinal and the occipital cortices (Raz, 2000; Squire et al., 2004; Raz et al., 2005; see Park and Reuter-Lorenz, 2009). These brain changes are associated with declines in processing speed, executive functions, working memory (WM) and episodic memory (e.g., Salthouse, 1996; Baltes and Lindenberger, 1997; Park and Gutchess, 2002; Nilsson, 2003; Rönnlund et al., 2005; Hoyer and Verhaeghen, 2006). General knowledge, verbal abilities (e.g., Park et al., 2002; see Hedden and Gabrieli, 2004; Craik and Bialystok, 2006), and implicit memory (e.g., Mitchell and Bruss, 2003; Wiggs et al., 2006) are mostly preserved. Even mild cognitively impaired older adults (Ballesteros et al., 2013b) and Alzheimer's disease patients (Ballesteros and Reales, 2004) showed preserved implicit memory despite deteriorations in episodic memory. Interestingly, electrophysiological (Osorio et al., 2010; Sebastián and Ballesteros, 2012) and event-related functional magnetic resonance imaging (Ballesteros et al., 2013a) studies have shown altered neural priming in older adults, despite preserved behavioral priming, suggesting a form of compensation.

Given the increase in life expectancy, and its association with occurrence of neurodegenerative diseases (e.g., Ferri et al., 2005; Brookmeyer et al., 2011; Reitz et al., 2011), identifying factors that might protect elders from cognitive decline is of great importance. Thus, the interest in interventions that can preserve and/or improve cognition in older adults has grown notably in the last decade. Computer-based interventions can easily be used with the elderly and could be a good alternative to traditional training programs (Kueider et al., 2012). Thus, researchers are increasingly using new technology tools, including cognitive training platforms and video games, to investigate their impact on cognition (e.g., Thompson and Foth, 2005; Craik et al., 2007; Mozolic et al., 2011; Buitenweg et al., 2012).

A wealth of data supports the view that there is potential for positive changes in older adults (e.g., see Hertzog et al., 2009; Valenzuela and Sachdev, 2009; Park and Bischof, 2013). Neuroplasticity, or the ability of the brain to adapt to environmental change by modifying neural connectivity and brain function (Knaepen et al., 2010), has been shown in animal studies, suggesting experience-induced increases in the hippocampus of those individuals living in an enriched environment (e.g., Kempermann et al., 2002, 2004).

Human studies have shown neural plasticity at several levels of the neural substrate (e.g., Pascual-Leone et al., 2005; Raz et al., 2005) although not to the same degree in old as in young adults (e.g., Bialystok and Craik, 2006; Li et al., 2006; Lee et al., 2008). The aging brain retains some neuroplasticity and the behavior of the individual can influence it (Cacciopo et al., 2006). The prolonged mismatch between functional organismic supplies and environmental demands produces cognitive plasticity and denotes the capacity of the brain for implementing behavioral flexibility (Lövdén et al., 2010; Bavelier et al., 2012). Based on the idea of neuroplasticity, different types of interventions have intended to ameliorate cognitive and functional decline by strengthening social networking (Waterworth et al., 2009; Peter et al., 2013), training cognitive skills (Jones et al., 2006), promoting an active lifestyle (Ballesteros et al., 2013c) or training physical activity (Colcombe et al., 2004; for reviews see Colcombe and Kramer, 2003; Hötting and Röder, 2013).

Training programs have been effective in improving older adults' cognitive performance in memory tasks (e.g., Craik et al., 2007; Smith et al., 2009; Hampstead et al., 2012) and other functions such as attention, working memory, reasoning, speed of processing, cognitive control and dual-task switching (e.g., Edwards et al., 2005; Bherer et al., 2006; Erickson et al., 2007; Berry et al., 2010; Mozolic et al., 2011; Anguera et al., 2013).

Training with video games is a fast-moving industry that claims to improve the cognition of users although the scientific evidence for this is at best mixed. A large-scale cognitive training study conducted by Ball et al. (2002) found that when memory, attention, and problem solving were trained independently, trainees improved in the skill trained but there was no transfer to other untrained skills. However, the main question is whether these benefits transfer to other untrained functions, improving the cognitive functioning of the elders (Lustig et al., 2009; see Buitenweg et al., 2012).

Cognitive training studies suggest that playing action video games enhances a variety of cognitive and perceptual abilities, including peripheral vision and visuospatial attention (e.g., Green and Bavelier, 2003, 2006; Green et al., 2010; Chisholm and Kingstone, 2012), visual short-term memory, switching between tasks, object mental rotation and executive control functions in young and older individuals (e.g., Basak et al., 2008; Boot et al., 2008; Colzato et al., 2010; Cain et al., 2012; Lee et al., 2012; for reviews see Bavelier et al., 2012). In contrast, other studies did not find transfer to cognitive functioning (e.g., Owen et al., 2010; Boot et al., 2013), improvements were very small (e.g., Ackerman et al., 2010), or young but not older adults showed transfer (e.g., Dahlin et al., 2008). For instance, Owen et al. (2010) trained 11,430 participants online for 6 weeks on cognitive tasks designed to improve planning, memory, reasoning, visuospatial abilities, and attention but they found no evidence that training improved cognitive functioning beyond the trained tasks.

Action video game training seems to improve several perceptual and cognitive abilities. However, most action video games are fast, intense, and unpredictable, emphasize peripheral processing, require selection between different action plans and might sometimes be violent (e.g., Green and Bavelier, 2003, 2006; Feng et al., 2007; Chisholm and Kingstone, 2012). These characteristics make this type of games unsuitable for older adults. Action games induced the lowest intervention compliance compared to non-action games. In addition, action games were rated as less enjoyable than non-action games (Nap et al., 2009; see McKay and Maki, 2010; Boot et al., 2013). Fortunately, studies with young (Oei and Petterson, 2013) and older adults (e.g., Cassavaugh and Kramer, 2009; Ackerman et al., 2010; McDougall and House, 2012; Nouchi et al., 2012; Anguera et al., 2013) suggest that cognitive improvements are not limited to action games.

Despite the interest in video games as intervention tools, evidence of their efficiency is at best mixed. However, a recent meta-analytic study (Toril et al., 2014) indicated positive effects that were moderated by variables such as the age of the trainees, the length of the training program, and the cognitive(s) function(s) assessed.

The present study was conducted to evaluate the effects of training older adults with non-action video games, to determine whether the benefits transfer to a broad number of cognitive functions. We trained a group of healthy older adults in the laboratory for 20 1-h sessions over the course of 10–12 weeks. In each session, the trainees practiced twice 10 non-action video games. We compared their pre- and post-test results in a series of psychological tests and computerized tasks with those of a non-contact control group to examine possible transfer of training to untrained tasks. The main question was whether the trained older brain would be able to use its plasticity to cope with age-related cognitive declines in several cognitive functions. A second goal was to find out whether the intervention would also improve the subjective wellbeing of the trainees.

## Materials and methods

The current study was approved and conducted in compliance with the guidelines set out by the *Universidad Nacional de Educación a Distancia* (UNED) Ethical Review Board. The study was conducted between January and July of 2013 in Madrid, Spain. All the participants gave their written informed consent before the study started and were remunerated 75€ for their participation. The remuneration was for travel expenses. They were informed of their right to terminate participation in the study at any time.

### Participants

Sixty participants were recruited through flyers, word of mouth, and local community centers for the elderly. Twenty of these declined to participate. Forty healthy older volunteers (age range 57–80-years-of-age) participated in the study. After signed the informed consent, they were randomly assigned to either the experimental group or the control group before being evaluated on the laboratory tasks. All participants lived active independent lives, with normal hearing and normal or corrected-to-normal vision. All reported being right-handed, and were free of neurological or psychiatric disorders and traumatic brain injury.

**Figure 1:**
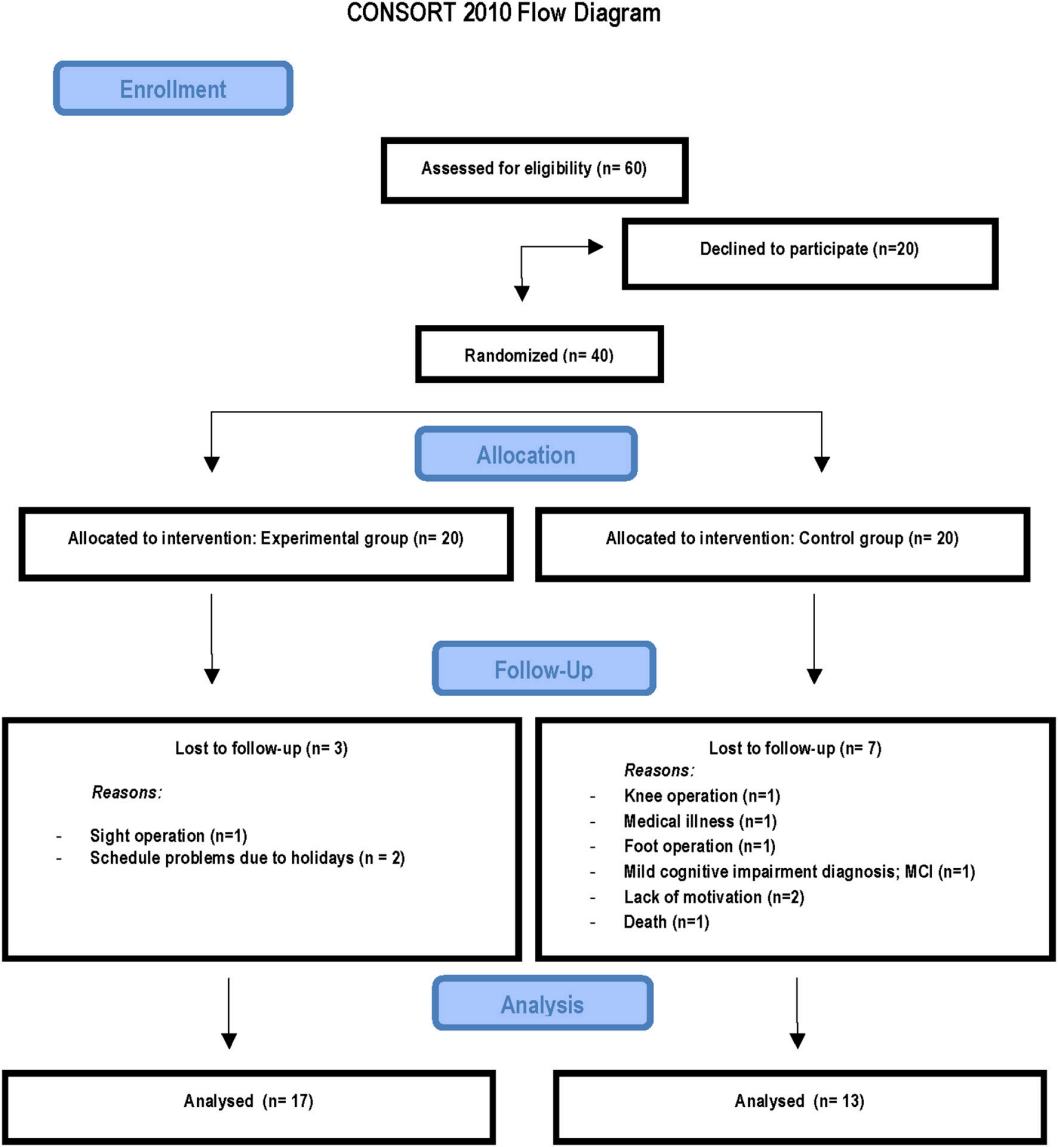
**Consort flowchart**.

The study was completed by 17 of the 20 participants in the video game training group (1 dropout suffered an eye operation during training and the other 2 dropouts had availability problems) and by 13 of the 20 participants in the control group (1 dropout had a knee operation, 1 had a foot operation, 1 was deceased, 1 was diagnosed with MCI during the course of the study, and 2 were not motivated). Analyses of background characteristics revealed no differences between participants remaining in the study and the dropouts within the respective group, except in the Mini-Mental State Examination and this was due to a control participant diagnosed with MCI during the course of the study (see Figure 1).

### Study design

To be included in the study, participants: (1) had to score 26 or above in the MMSE (Folstein et al., 1975); (2) obtain under 5 in the Yesavage Depression Scale (Yesavage et al., 1983; Spanish adaptation by Martínez et al., 2002); and (3) obtain a normal score in the Vocabulary subscale of the WAIS-III scale (Wechsler, 1999). Table 1 summarizes the demographics and screening test scores for each group. *T*-tests showed no significant differences between the two groups (all *ps* > 0.05) for all of these measures prior to the intervention.

**Table 1:** **Demographic Information for participants in each group**.

**Characteristic**	**Experimental**	**Control**	**η^2^_*p*_**	***p***	***F***
Women/men (n)	10/7	8/5	0.001	0.885	0.021
Age (years)	68.8 (5.15)	69.2 (5.91)	0.001	0.849	0.037
Education (years)	12.2 (5.09)	12.9 (3.28)	0.008	0.649	0.212
MMSE	28.7 (1.16)	28.8 (1.03)	0.001	0.847	0.038
Depression	1.5 (1.18)	2.4 (2.88)	0.035	0.320	1.023
Verbal ability	62.4 (9.43)	60.8 (7.37)	0.017	0.495	0.479

*Means and Standard deviations (SDs in parentheses); MMSE, Mini-Mental State Examination; Verbal ability, Vocabulary (Wechsler); Depression, Yesavage Depression Scale*.

Transfer of training was measured as performance improvement at post-test relative to pretest. Participants in both groups individually completed a pretest assessment distributed over 2 days (2-h per day). Immediately following the intervention phase, or a similar period in the case of the controls, all participants completed two post-test sessions similar to pretest. Participants in both groups attended the 5 assessment sessions (screening, 2 pre-, and 2 post-test sessions). At pretest (session 1), participants performed a cross-modal oddball task, followed by the spatial WM tasks (Rey-Osterrieth Figure Test, Corsi Blocks and Jigsaw-Puzzle Test). After a rest, they performed the Wisconsin Card Sorting Task (WCST), between two speeded tasks (simple and choice RT tasks) that were counterbalanced across participants. In session 2, participants performed Memory Faces 1 and Wellbeing IPF-IL followed, after a 25 min rest, by Memory Faces II. Then, participants performed Family Pictures I followed, after 25 min by Family Pictures II. The post-test was identical to the pretest.

### Overview of the training program

Participants assigned to the experimental group underwent 20 1-h training sessions over 10–12 weeks. In each session, they practiced twice 10 video games selected from *Lumosity* (http://www.lumosity.com), a web-based cognitive training platform that includes games designed with the purpose of improving the user's cognitive abilities (Stemberg et al., 2013). Participants practiced the games in our laboratory on a PC equipped with a 21-inch monitor. Scores on each game were recorded. Below we describe the games briefly. The control group did not receive training but met three times with the researchers in a room of the laboratory. Each meeting lasted about 2 h. During these meetings members of the research group discussed with the participants general topics related to aging and their interests. They have coffee and soft drinks together.

#### Speed mach

The player was presented with a symbol that appears on the computer screen and had to remember the symbol and compare it with the symbol that appeared immediately before (same, different) by pressing one of two keys as soon as possible.

#### Memory matrix

A variable in size matrix appeared at the center of the screen with a pattern of blocks temporarily displayed. The player reported the position of the blocks by clicking on the positions of the matrix where the blocks were presented.

#### Rotation matrix

An array of blocks rotated and the player had to rotate mentally the pattern and click on the correct positions.

#### Face memory

The player had to decide whether the face that currently appeared on the screen matched the face shown one (1-back), two (2-back), or three (3-back) faces shown before.

#### Memory match

Players had to remember each new symbol and compare it with the one shown previously (same, different) by pressing one of two keys as fast as possible.

#### Money comb

A money comb appeared at the center of the screen and a sequence of tokens of different values was presented briefly inside it. The task consisted of clicking on the correct tiles to reveal the tokens in the correct order (from lowest to highest value).

#### Lost in migration

A static flock of birds appeared at the center of the screen. The goal was to identify the orientation of the bird that appeared at the center by pressing one of the four arrow keys on the keyboard as fast as possible.

#### Space junk

After a brief countdown, a series of elements appeared on the screen. The player determined the number of the presented items by pressing the corresponding number on the screen number pad.

#### Raindrops

A raindrop containing an equation was falling down from the top of the screen. The gamer had to solve the equation before it reached the water below by pressing the correct number on the number pad.

#### Chalkboard

Two boxes with different numbers appeared on the screen. The gamer determined which box contained the larger number by pressing the arrow keys on the keyboard.

Participants received points based on their performance on each video game. In four games (*Memory match, Memory faces, Speed match, and Lost in migration*) the time necessary to complete the game was also recorded. None of the participants in the study informed of having any previous experience with video games.

### Assessment tasks and procedures

Assessment measures fell into one of six broad domains: processing speed, attention, executive control, spatial working memory, episodic memory, and subjective wellbeing. The computerized tasks (simple and choice reaction time), oddball, Corsi blocks, Jigsaw-puzzles) were programmed using E-Prime 2.0 (Psychology Software Tools Inc, Pittsburg, PA, USA). Executive control was assessed with a computerized version of the Wisconsin Card Sorting Test (WCST). To perform these tasks, participants were comfortably seated at a distance of approximately 55 cm from the computer screen. These tasks and psychological tests are described below.

### Speed of processing (simple and choice RT) tasks

Task order was counterbalanced across participants. Each task started with a practice block with visual feedback followed by 4 blocks of 40 trials each. In the simple RT task, participants viewed a target that appeared at the center of the computer screen in Times New Roman font (size 20) and pressed a designated key as soon as possible. Each trial consisted of a fixation cross (1000 ms), followed by a blank screen displayed for 500 or 1000 ms (randomly selected) after which the target (“X” in the simple RT task) appeared. In the choice RT task the stimuli were “X” or “O” and participants pressed a designated key for each of them. Response keys were counterbalanced across participants. Stimuli disappeared after response or after 5000 ms. The inter-trial interval lasted 750 ms. In 10 percent of the trials, the target was not presented (catch trials) in the detection task. The dependent variable was response time for correct responses. Both tasks lasted approximately 15–20 min and all participants completed a practice session with visual feedback before the start each RT task.

### Executive control (WCST)

Participants performed the WCST (CV4; Heaton, 1981). On each trial, four cards differing in shape (square, triangle, circle or star), color (blue, red, yellow or green) and number of shapes (one, two, three, or four) were displayed on the computer screen. The participant sorted the cards into different categories according to shape (S), color (C), or number of shapes (N). The task consisted on matching a card displayed in the lower right corner of the screen with one of the four cards displayed at the top. Identification of the sorting category was based on feedback from the computer. The sorting criterion changed after 10 consecutive correct trials. Each response card could be matched with a stimulus card on the basis of one or more dimensions. The computer recorded each response as correct or incorrect. The task ended after 10 consecutive responses to each category in the order CSNCSN, or when the two sets of 64 response cards had been presented. The main dependent variable was the number of perseverative errors, which is associated with the shrinking of frontal areas (Gunning-Dixon and Raz, 2003). Patients suffering from prefrontal brain lesion persist in using the same rule (Milner, 1963). The task lasted approximately 30–40 min.

### Cross-modal oddball attention task

We used an in-house developed cross-modal visual-auditory oddball task to assess distraction and alertness. Participants categorized a visual digit from 1 to 8 as odd or even by pressing one of two keys (counterbalanced across participants). There were 3 blocks of 384 trials each. A trial began with the presentation of a fixation cross at the center of the screen as well as a 200 ms sound. The digit appeared 100 ms after the sound's offset, and remained on the screen for 200 ms. There were 3 sound conditions: A silent block and two block of trials containing two different sounds, the standard sound (used in 80% of the trials) that was a 600 Hz sine wave tone of 200 ms, and the novel sound (the 20% of the trials; e.g., drill, hammer, rain). Sounds were presented binaurally through headphones at approximately 75 dB SPL. Results from this task have been reported separately (see Mayas et al., 2014).

### Visuospatial working memory

Spatial WM (Baddeley and Hitch, 1974), was assessed with the *Corsi blocks* and the *Jigsaw-puzzle tasks*. Participants also performed the *Rey-Osterrieth Complex Figure Test*.

#### Corsi task

The original task (Milner, 1971) consisted of a set of nine identical blocks (3 × 3 × 3 cm) unevenly positioned on a wooden board (23 × 28 cm). The participant had to point to the blocks in their presentation order. The length of the block sequences increases until recall was no longer correct. We used a computerized version of the task with four difficulty levels (2, 3, 4, and 5 cubes) and 10 trials per level. The stimuli appeared one by one at the computer screen inside a 10 × 10 cm matrix for 1000 ms each. On each trial, the participant reproduced the pattern of cubes just presented. The score was the proportion of correct sequences for each level.

#### The Jigsaw-puzzle task

The pencil and paper task was developed to assess active visuospatial abilities (Richardson and Vecchi, 2002). In our computerized version, the puzzles consisting of 4, 6, or 9 pieces were presented at the computer screen. Each piece was numbered and the participant had to write down the number corresponding to the pieces in the correct spatial positions. To our knowledge, this is the first computerized version of the task. The stimuli were 15 pictures (e.g., kettle, lamp, chair) with similar visual complexity selected from Snodgrass and Vanderwart (1980). Each picture was fragmented into four, six and nine pieces to produce 45 different puzzles. The pictures were enlarged to fit an area 12 × 12 cm and divided into four pieces of 6 × 6 cm, six pieces of 6 × 4 cm, or nine pieces of 3 × 3 cm. Three different counterbalanced orders were generated. Different pictures were used at pre and post-testing. Participants viewed 15 puzzles representing all possible combinations of visual complexity and number of pieces. The response sheets contained grids of the same size as the original pictures with the appropriate number of squares (4, 6, 9, squares). We used 2 puzzles as practice and their results were not included in the analysis. In each trial, a fragmented picture appeared on the screen and the participant wrote down in the response sheet the appropriate numbers to form a spatially correct picture. The jigsaw was presented on the computer screen for 90 s. Participants were allowed to correct errors within this time. The proportion of correct puzzles per level (4, 6, and 9 pieces) was the dependent variable.

### Rey-Osterrieth complex figure test

We used the Spanish adaptation of the test (Rey, 1942, 1999) to assess visual constructive abilities and visuospatial memory. Participants reproduced a complex drawing, first by copying it and then by reproducing the drawing from memory.

### Immediate and delayed visual episodic memory

Immediate recognition memory for Faces and Family Pictures were assessed with Faces I, and Family Pictures I, while delayed memory (25 min after encoding) was assessed with Faces II and Family Pictures II, from the Spanish version (Wechsler and Pereña, 2004) of the Memory Wechsler Scale, WMS-III (Wechsler, 1997).

### Wellbeing

We assessed wellbeing with the 15-item short version of the SPF-IL Scale (Nieboer et al., 2005) which assesses five wellbeing dimensions. Subjective wellbeing is the overall state of wellbeing of a person determined by his/her ability to obtain the goals of physical and social wellbeing. The maximum score per dimension was 12. The *Affection* dimension relates to the degree of confidence and social acceptance and their level of satisfaction with the people around them. *Assertivity* refers to the self-perception to have done the right thing in the eyes of relevant others. *Status* assesses the feeling of being treated with respect, self-realization, achievement as compared to others, and reputation. *Comfort* is the absence of feelings of discomfort, pain or stress. Finally, *Stimulation* refers to mental and physical activation.

## Results

The main question examined was whether training with non-action video games enhanced cognition of older adults and if training would transfer to other untrained broader cognitive functions. The question was tested by considering whether group (control group, video game trained group) interacted with testing session (pre, post-testing) with regard to performance on a series of cognitive measures.

### Video game performance

**Figure 2:**
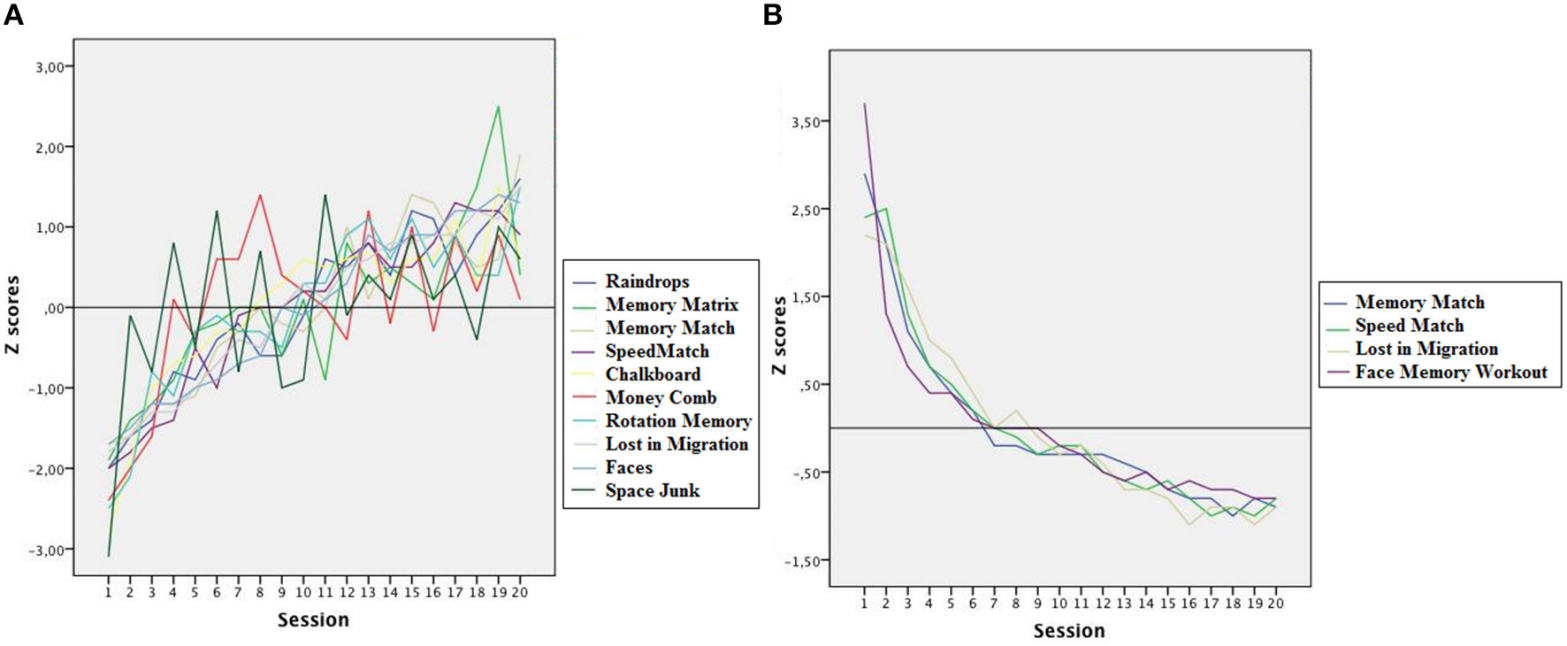
**(A)** Average performance scores obtained in each of the 10 non-action video games across the 20 training sessions in Z scores (mean 0; standard deviation 1). **(B)** Average response times of 4 video games across the 20 training sessions in Z scores.

Video game performance showed significant improvements across sessions. The mean performance on each game was compared at the beginning and the end of training using regression analysis with *Training Session* as the predictor variable and *Reaction Time* and *Game Score* as the criterion variables. The results showed that performance in all games improved significantly after training. *R^2^* coefficients were high and accounted for more than 80% of the variance of the model in all games except *Memory Matrix*, *Money Comb*, and *Space Junk*. The ANOVAs for the previous analyses indicated that all *R^2^* coefficients were significant, so Training Session was a reliable predictor of Score and RT in all games. Table 2 presents a summary of the results and Figure 2 shows the improvements in each video game as a function of training session in Z scores.

**Table 2:** **Determination coefficients (*R*^2^), *F* and *p*-values for the 10 trained video games**.

**Video game**	**DV**	***R*^2^ (corr.)**	***F* (*p*)**
Speed match	Score	0.961 (0.959)	441.77 (0.000)
	RT	0.965 (0.963)	492.66 (0.000)
Memory matrix	Score	0.698 (0.681)	38.60 (0.000)
Rotation matrix	Score	0.884 (0.878)	137.32 (0.000)
Money comb	Score	0.601 (0.579)	27.15 (0.000)
Face memory	Score	0.972 (0.971)	626.61 (0.000)
	RT	0.977 (0.976)	719.10 (0.000)
Memory match	Score	0.852 (0.844)	103.81 (0.000)
	RT	0.960 (0.958)	430.21 (0.000)
Lost in migration	Score	0.964 (0.962)	484.67 (0.000)
	RT	0.965 (0.963)	500.39 (0.000)
Space junk	Score	0.485 (0.457)	16.97 (0.001)
Raindrops	Score	0.893 (0.887)	150.88 (0.000)
Chalkboard challenge	Score	0.919 (0.914)	203.60 (0.000)

*DV, (dependent variable); R^2^ (Regression coefficient); corr. (corrected regression coefficient); F (F-values of ANOVAs); p (significance)*.

### Effects of the intervention program on cognitive performance

Next, we explored whether training led to enhancements of cognitive abilities that deteriorate with age. We conducted analyses of variance (ANOVAs) with group (experimental, control) and session (pre, post-testing) as independent variables. Group was inter-subjects while Session was considered as repeated intra-subjects. Analyses were conducted using the SPSS (Version 21).

### Simple and choice RT tasks

Mean results and standard deviations are displayed in Table 3. Incorrect trials (less than 1% for both groups) and those with RTs faster than 200 ms or slower than 1500 ms (0.7 and 1.19% for experimental and control group, respectively) were excluded from RT analyses.

**Table 3:** **Pre and post-training performance on psychological measures and wellbeing dimensions for the experimental and control groups**.

		**Experimental group**		**Control group**	
		**Pre**	**Post**	**Pre**	**Post**
Speed of processing	Detection (ms)	460 (74)	483 (76)	466 (69)	464 (75)
	Choice RT (ms)^**^	596 (106)	566 (80)	570 (62)	578 (86)
Cross modal oddball task	Distraction (ms)^**^	40.1 (34.0)	27.9 (25.5)	38. 6 (31.2)	37.8 (27.7)
	Alertness (ms)^**^	7.4 (24.7)	32.6 (35.8)	25.3 (32.9)	17.6 (45.5)
WCST	Error (%)	34.9 (15.5)	29.4 (18.1)	36.0 (17.9)	30.7 (16.2)
	Perseverative Resp (%)	18.0 (12.6)	16.9 (14.3)	17.8 (9.7)	14.3 (8.3)
	Perseverative Error (%)	15.7 (9.3)	15.2 (11.3)	16.4 (8.7)	13.5 (7.0)
	Non-Persev. Error (%)	15.3 (8.5)	14.1 (12.8)	19.6 (8.3)	17.3 (12.9)
	Conceptual Level (%)	58.3 (23.1)	63.5 (23.6)	52.5 (25.3)	60.3 (22.1)
Jigsaw puzzle task	4 Pieces (proportion)	0.78 (0.26)	0.75 (0.28)	0.78 (0.22)	0.63 (0.28)
	6 Pieces (proportion)	0.40 (0.25)	0.52 (0.48)	0.35 (0.33)	0.43 (0.28)
	9 Pieces (proportion)	0.01 (0.05)	0.07 (0.17)	0.05 (0.09)	0.14 (0.21)
Corsi blocks task	2 Serial Position (proportion)	0.96 (0.07)	0.98 (0.05)	0.95 (0.07)	0.98 (0.04)
	3 Serial Position (proportion)+	0.88 (0.16)	0.80 (0.25)	0.72 (0.26)	0.85 (0.13)
	4 Serial Position (proportion)	0.71 (0.20)	0.73 (0.27)	0.52 (0.21)	0.54 (0.25)
	5 Serial Position (proportion)	0.32 (0.22)	0.36 (0.23)	0.28 (0.24)	0.22 (0.17)
Rey	Copy	31.9 (3.1)	33.2 (4.6)	30.2 (3.3)	34.4 (0.87)
	Delayed Recall	17.2 (6.6)	19.1 (6.6)	16.9 (3.5)	20.2 (7.2)
WMS-III	Faces (Immediate)	35.9 (4.7)	39.0 (4.5)	36.8 (4.0)	39.3 (4.0)
	Faces (Delayed)	35.5 (4.1)	37.9 (5.2)	37.0 (3.4)	38.8 (5.6)
	Family Pictures (Immediate)^**^	29.8 (6.3)	35.5 (8.1)	31.2 (7.3)	29.2 (9.6)
	Family Pictures (Delayed)^**^	27.6 (6.5)	35.8 (8.5)	26.6 (6.0)	28.0 (9.1)
SPF-IL	Affect ^*^	8.9 (1.8)	9.8 (1.7)	9.4 (2.3)	9.0 (1.7)
	Assertiveness^*^	9.4 (1.7)	10.5 (1.3)	9.6 (1.1)	9.7 (1.4)
	Status	7.5 (1.8)	8.4 (1.7)	7.9 (1.2)	7.8 (1.8)
	Comfort	8.4 (1.5)	9.2 (2.1)	8.7 (1.5)	9.4 (1.9)
	Stimulation	8.8 (1.3)	8.8 (1.6)	7.8 (1.1)	7.8 (1.7)

Mean scores of the outcome measures, with standard deviation in parentheses. WCST (Wisconsin Card Sorting Test); REY (Rey-Osterrieth Complex Figure Test); WMS-III (Wechsler Memory Scale); Dimensions of wellbeing (SPF-IL Scale). Double asterisk (

** ) indicates tests on which the experimental group showed significant greatly improvements than controls (p < 0.05), single asterisk (

* *) indicates the test on which there was a trend for larger improvements in the experimental group (p < 0.10), and the cross (+) indicates the test on which control group showed significantly greater improvements than the experimental group (p < 0.05)*.

**Figure 3:**
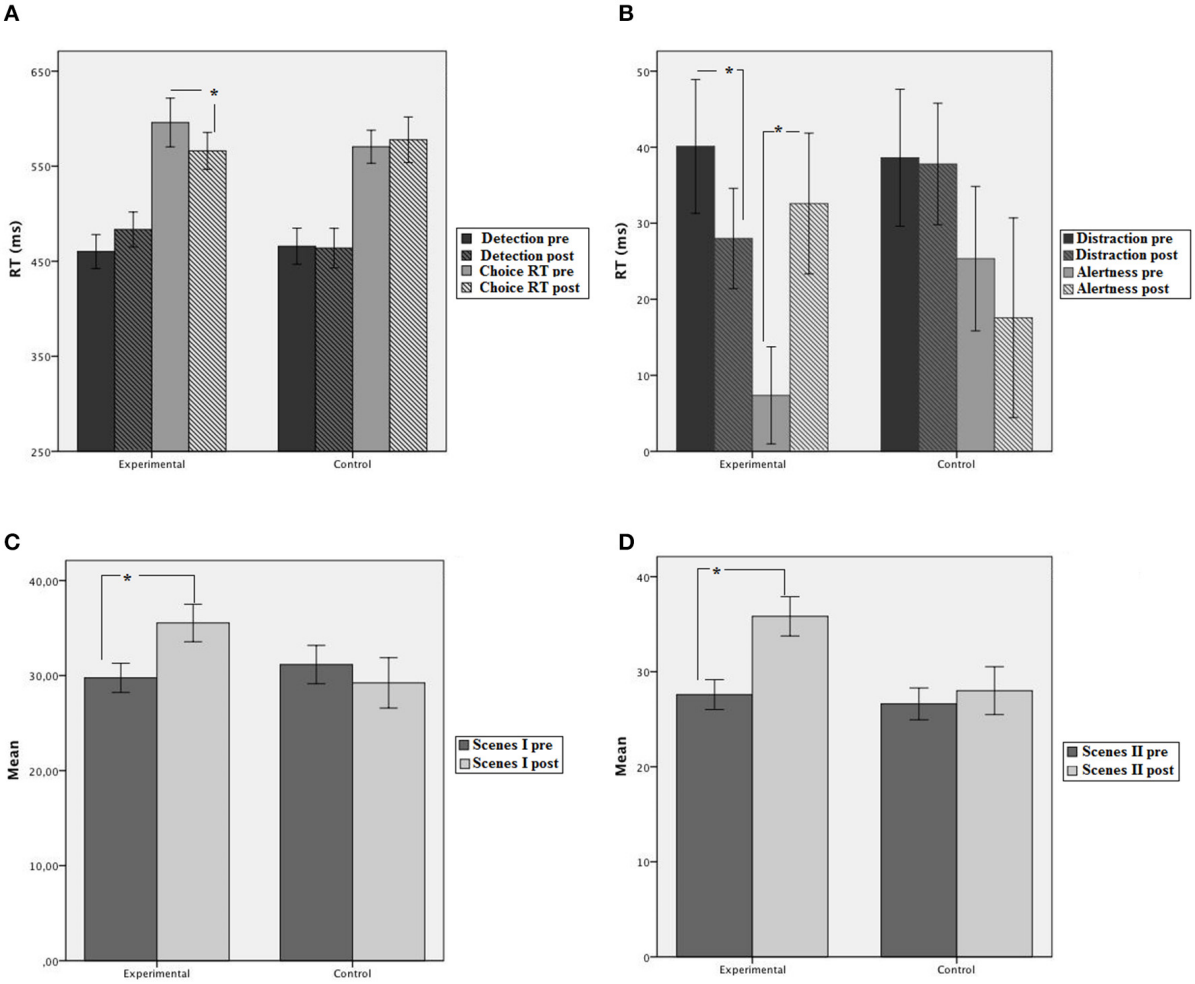
**Mean performance of trained and control groups at pretest and post-test. (A)** Simple and choice RT tasks. **(B)** Distraction and alertness effects in the Cross-modal oddball attention task. **(C)** Family pictures (Scenes) inmediate. **(D)** Family pictures (Scenes) delayed. Error bars represent plus and minus 1 standard error. ^*^p < 0.05.

A 2 group (experimental, control) × 2 session (pretest, post-test) × 2 task (detection, choice) mixed ANOVA was conducted on mean RTs. The main effect of task was statistically significant [*F*_(1, 28)_ = 144.11, *MSE* = 2440.89, *p* < 0.001, η^2^_*p*_ = 0.83]. Participants performed faster on the detection task (468 ms) than the choice RT task (578 ms). No other main effect was significant (*ps* > 0.05). However, the three-way interaction group × session × task was statistically significant [*F*_(1, 28)_ = 3.24, *MSE* = 1812.22, *p* = 0.057, η^2^_*p*_ = 0.12]. The interaction showed that groups did not differ at pre and post-training in the detection task (460 and 483 ms for the experimental group; 466 and 464 for the control group in pre and post-test, respectively). However, in the choice RT task, the experimental group improved performance after training (566 and 496 ms for pre and post-session, respectively) while the control group did not (570 and 578 ms for pre and post, respectively; see Figure 3).

### Executive functions (WCST)

Table 3 shows the main dependent variables of WCST for both groups at pre and post-tests. Separate mixed ANOVAs with group and session were conducted with percentage of errors, percentage of perseverative responses, percentage of perseverative errors, percentage of non-perseverative errors and percentage of conceptual level errors. The main variable of interest was the proportion of perseverative errors; that is, the total number of errors that occur when the participant is required to switch to another sorting rule but instead, the participant persists in sorting using the rule that was previously correct, relative to the number of trials administered multiplied by 100.

A group × session mixed ANOVA was performed with the percentage of perseverative errors as the dependent variable. Neither the main effect of group [*F*_(1, 28)_ = 0.02, *MSE* = 150.26, *p* > 0.05, η^2^_*p*_ = 0.001] or session [*F*_(1, 28)_ = 1.59, *MSE* = 26.85, *p* > 0.05, η^2^_*p*_ = 0.05], nor the interaction group x session were significant (*F*_(1, 28)_ = 0.91, *MSE* = 26.85, *p* > 0.05; η^2^_*p*_ = 0.03; see Table 2). The same pattern of results was obtained in the ANOVAs conducted in the other dependent measures (all *p*s > 0.05).

### Cross-modal oddball attention task

Accuracy levels in the digit categorization task pre-post in both groups were similar [*F*_(1, 25)_ = 1.38, *MSE* = 0.05, *p* > 0.05, η^2^_*p*_ = 0.05]. Mean accuracy was 0.90 and 0.92 at pre-post for the experimental group, and 0.90 and 0.90 at pre-post for the control group. The main effect of condition was significant [*F*_(2, 50)_ = 5.52, *MSE* = 0.001, *p* < 0.005, η^2^_*p*_ = 0.18], showing lower correct responses in the novel compared to the silent and the standard conditions [*F*_(1, 25)_ = 7.087, *MSE* = 0.001, *p* < 0.05, and *F*_(1, 25)_ = 5.99, *MSE* = 0.0005, *p* < 0.05, respectively] while the standard and silent conditions did not differ from each other (*p* = 0.150). The effect of session was not significant [*F*_(1, 25)_ = 2.25, *MSE* = 0.0025, *p* < 0.15, η^2^_*p*_ = 0.08]. None of the two-way interactions were significant (all *p*s > 0.05). The ANOVA performed on correct response times after removing outliers with group and session as between-subjects and condition (silence, standard and novel) as the within-subjects factor, revealed that group was not statistically significant [*F*_(1, 25)_ = 1.2, *MSE* = 40075.19, *p* = 0.27) but the main effect of condition was significant [*F*_(2, 50)_ = 26.63, *MSE* = 657.99, *p* < 0.001, η^2^_*p*_ = 0.51]. The mean RT corresponding to the standard condition was the fastest (613 ms), while the slowest was the RT corresponding to the novel condition (649 ms). The main effect of session was not significant (*p* > 0.05). However, the triple interaction group × session × condition was statistically significant [*F*_(2, 50)_ = 4.37, *MSE* = 403.54, *p* < 0.05; η^2^_*p*_ = 0.51]. New analyses were conducted on distraction (novel *vs*. standard sound) and alertness (silence vs. standard sound), showing that the ability to ignore relevant sounds (distraction) improved significantly in the experimental group after training (12 ms) but not in the control group. The analyses of alertness showed that the experimental group increased 26 ms in alertness (*p* < 0.05) but control group did not (*p* = 0.54).

### Spatial working memory (WM)

#### Corsi blocks

A group (2) × session (2) x number of blocks (4: 2, 3, 4, and 5 blocks) mixed ANOVA with group as the between-subject factor and time and number of blocks as the within-subject factors was performed on the proportion of correct sequences [number of correct sequences per level/number of sequences per level]. Group and session were not significant (all *p*s > 0.05) but number of blocks was significant [*F*_(1, 28)_ = 237.415, *MSE* = 4.98, *p* < 0.001, η^2^_*p*_ = 0.89]. Performance deteriorated with the increase of the number of blocks. The interaction group x number was significant [*F*_(1, 28)_ = 4.35, *MSE* = 0.09, *p* < 0.001, η^2^_*p*_ = 0.89], suggesting better performance of the experimental group in the level 4 of the Corsi task (*p* = 0.013) and no difference in the other block conditions. Moreover, the triple interaction group × session × number was statistically significant [*F*_(1, 28)_ = 3.98, *MSE* = 0.06, *p* < 0.01, η^2^_*p*_ = 0.12]. The interaction was due to a different pattern of performance between groups with 3 blocks. While the experimental group performed worse at post-test than pretest, an opposite pattern was found in the control group (*p* < 0.05). No other effect was significant.

#### Jigsaw-puzzle task

The ANOVA conducted with group, session, and level (4, 6, and 9 pieces) showed that group and session were not statistically significant (*ps* > 0.05) but level was [*F*_(1, 28)_ = 121.352, *MSE* = 6.61, *p* < 0.001, η^2^_*p*_ = 0.89]. Performance decreased as the number of pieces increased from 4 to 9 pieces. The interaction session x level was significant [*F*_(1, 28)_ = 3.40, *MSE* = 0.15, *p* = 0.04, η^2^_*p*_ = 0.10], suggesting that participants performed better at post-test with jigsaws of 6 (0.40 and 0.48) and 9 pieces (0.02 and 0.10) and did not differ with jigsaws of 4 pieces.

### Visual episodic memory

#### Faces

A mixed ANOVA group x session x delay (immediate, delayed) was performed on the recognition scores. The analysis showed a significant effect of session [*F*_(1, 28)_ = 17.98, *MSE* = 176.74, *p* < 0.001, η^2^_*p*_ = 0.39]. The recognition score was significantly higher at post-test (mean = 38.76) than at pretest (mean = 36.31 and *SD* = 3.76). No other main factor or interaction was significant.

#### Family pictures

The group × session × delay mixed ANOVA showed a significant main effect of session [*F*_(1, 28)_ = 4.64, *MSE* = 333.73, *p* < 0.05, η^2^_*p*_ = 0.14] and delay [*F*_(1, 28)_ = 22.78, *MSE* = 107.82, *p* < 0.001, η^2^_*p*_ = 0.45]. The interaction group × session was significant [*F*_(1, 28)_ = 5.41, *MSE* = 389.27, *p* < 0.05, η^2^_*p*_ = 0.16]. Groups did not differ at pretest but at post-test the experimental group showed better performance compared to pretest (35.65 vs. 28.68) while the control group did not improve (28.62 vs. 28.86). The interactions group x delay [*F*_(1, 28)_ = 5.88, *MSE* = 27.82, *p* < 0.05, η^2^_*p*_ = 0.174] and session × delay were significant [*F*_(1, 28)_ = 13.02, *MSE* = 61.49, *p* < 0.01, η^2^_*p*_ = 0.32]. As these interactions did not include the group factor, they are not discussed further.

### Wellbeing

A 2 × 2 mixed repeated measures ANOVA was conducted for results on each of the 5 subscales of the SPF-IL, with group as the between-subjects factor and session as the within-subjects factor. The results showed a main effect of session in *Assertiveness* [*F*_(1, 28)_ = 4.41, *MSE* = 5.26, *p* < 0.05, η^2^ = 0.14) and *Comfort* [*F*_(1, 28)_ = 5.08, *MSE* = 8.46, *p* < 0.05, η^2^ = 0.15] subscales, due to a general increase in scores at post-test. Group reached statistical significance in the *Stimulation* subscale [*F*_(1, 28)_ = 5.58, *MSE* = 13.24, *p* < 0.05, η^2^ = 0.17], indicating higher scores in the experimental relative to the control group. The interaction group × session was marginally significant for *Affection* [*F*_(1, 28)_ = 3.42, *MSE* = 6.47, *p* = 0.07, η^2^ = 0.11] and *Assertiveness* [*F*_(1, 28)_ = 3.35, *MSE* = 3.99, *p* = 0.08, η^2^ = 0.11] subscales. Planned comparisons showed that the trend was due to the improvement of the experimental group after training suggesting that the trained group improved (marginally) in these dimensions after training while the control group did not.

## Discussion

The results confirmed that the trainees improved significantly in the practiced games (e.g., Ackerman et al., 2010; Reddick et al., 2013). This study yielded two main findings. First, the results indicated that the trained group showed enhancements compared to control participants in: (a) controlled processing, as shown by the significant improvement in the choice RT task; (b) attention, as the trainees were less distracted by irrelevant sounds and showed an increase in alertness; (c) immediate and delayed recall memory for family pictures (WMS-III); and (d) *Affection and Assertiveness* subscales of the Wellbeing scale. Given the age-related declines in these functions, the results seem encouraging. Second, our trained participants neither showed transfer to executive control assessed with the WCST nor to spatial WM assessed with three different tasks.

### Non-action video game training transferred to cognition and wellbeing

The enhancement found in processing speed agreed with previous results (e.g., Drew and Waters, 1986; Clark et al., 1987; Maillot et al., 2012). This result has implications for applied aging science, as processing speed is a robust predictor of age-related cognitive decline (e.g., Salthouse, 1996; Zimprich, 2002; Salthouse and Ferrer-Caja, 2003; Eckert, 2011). Speed of processing training is an indicator of efficient performance of daily living activities and safer driving performance (e.g., Ball et al., 2007; Wolinsky et al., 2013).

We also found that the trainees reduced distractibility by improving alertness and attention filtering, functions that decline with age and largely depend on frontal regions (Mayas et al., 2014). A marginal improvement after training was observed in *Affection* and *Assertiveness*, two dimensions of subjective wellbeing. The first relates to the degree of confidence and social acceptance and their level of satisfaction with the people around them while the second indicates the self-perception of doing good things, be a good person, and contribute to a common goal. Other studies found no significant changes after training either on self- reported mood and mental health state (Mozolic et al., 2011) or in real-world outcomes (Willis et al., 2006).

### Training did not enhance executive control and spatial WM

The frontal lobe hypothesis assumes that age-related cognitive declines are the results of changes that occur in the frontal lobes (West, 1996; Raz, 2000). Older adults are less efficient in inhibitory functioning than younger adults (see Hasher et al., 1999; Andrés et al., 2006). The executive functioning comprises several cognitive abilities including resistance to interference, memory updating, and shifting (Miyake et al., 2000) and decline with age (Kray and Lindenberger, 2000). Larger age-related declines in all WCST indexes were found in MCI older adults compared to healthy elders (Ballesteros et al., 2013b) while a long-term physically active lifestyle improved older adults' performance on the WCST compared to sedentary older adults (Ballesteros et al., 2013c).

Our results did not agree with those of Basak et al. (2008) who found improvements in executive control after training older adults with a video game that combines the speed of real-time gaming with the complexity of strategy-based video games. Possibly, to improve task shifting, it would be necessary to train with strategy-based video games. However, Boot et al. (2013) trained a group with an action game (*Mario Kart DS*) and other group with a non-action game (*Brain Age 2*) and did not found any improvement.

Our findings agree with two recent meta-analyses conducted with older adults (Toril et al., 2014) and with trainees from all ages (Powers et al., 2013). Both studies found negligible effects of video game training on executive functions. Only first-person shooter action games studies have reported benefits after training (Green and Bavelier, 2003, 2006). These types of games require great perceptual abilities and a large emphasis on peripheral visual processing (Green et al., 2010). However, older adults do not like this type of games (Nap et al., 2009; see Boot et al., 2013). Our results contrast with those of Nouchi et al. (2012) who found improvements on executive functions after training older adults with *Brain Age 2* after just 5 h of training and Maillot et al. (2012) with exergames training.

The ineffectiveness of training despite the fact that several of the non-action video games mimic WM tasks agreed with findings from two recent reviews (Shipstead et al., 2012; Melby-Lervåg and Hulme, 2013). Importantly, a recent randomized, placebo-controlled study with young adults who received 20 sessions of practice on an adaptive computerized *n-back* training program found no evidence of improvement in multiple measures of verbal and spatial fluid intelligence, multitasking, or WM capacity (Reddick et al., 2013). Boot et al. (2013) reported similar results in Corsi blocks.

## Conclusions, limitations and future directions

Animal (e.g., Chen and Tonegawa, 1997; Holtmaat and Svoboda, 2009) and human studies (e.g., Colcombe et al., 2004; Pascual-Leone et al., 2005; Lindenberger et al., 2006; Anguera et al., 2013) have shown that the adult brain maintains some plasticity, which allows behavior modification through practice. Cognitive training programs based on repetitive practice on cognitive processes have been effective in improving the trained process but not so much other untrained cognitive functions (e.g., Mahncke et al., 2006; Ball et al., 2007; Mozolic et al., 2011; Hampstead et al., 2012).

Our results revealed a causal effect of game training on the cognitive enhancement of healthy elders. Boot et al. (2013) found that neither a group of elders trained with an action video game, nor another trained with a non-action game improved on any cognitive ability compared to a control group. It is possible that the negative results were due to the long training regime (50 h vs. 10 to 20 h in other studies) as the effects are greater when training is of short duration (1–6 weeks) than when it is long (7–12 weeks) in old people (Toril et al., 2014). This finding has practical implications as many intervention programs spend a great amount of time training older participants, on the assumption that longer training will produce better results.

The current study had relatively small sample sizes as have other recent randomized training studies (e.g., Mozolic et al., 2011; Hampstead et al., 2012; Nouchi et al., 2012). Despite the relative small sample used in the present study, we found that participants that completed the 20 training sessions were faster on a choice RT task, showed reduced distraction and an increase in alertness on an oddball attention task, improved memory for visual stimuli and improved (marginally) in two dimensions of wellbeing. These results suggest that the brain of the elderly retains certain neurocognitive plasticity. Whether similar changes in these cognitive functions after training with video games might be observed in young adults, who are at the peak of cognitive functioning, is an important and open question that would deserve further investigation.

It would also be important to demonstrate in future studies maintenance of gains in speed of processing, attention, immediate as well as delayed visual recognition memory and variables of wellbeing using long follow-up periods. Future analyses should inform whether cognitive and wellbeing gains are maintained over time after finishing the training program. Our study presents other limitations. One is that it does not examine whether the effect of training may generalize to everyday life tasks. Future studies should investigate whether the improvements found would transfer to real world tasks. Another limitation is related to the difficulty to rule out possible effects due to motivational factors, the familiarity or confidence with the researchers rather than neurocognitive plasticity as the trained group had more contact with the experimenter than the control group. Hence another possible avenue for research will be to include two types of control groups, an active control group and a no-contact control group. However, it should be mentioned that in a recent meta-analytic study (Toril et al., 2014), we have analyzed the effect sizes of those published studies that used both an active and a passive control group (excluding those studies that had just a passive control or no control group). The difference between active and passive control was not statistically significant but they were significantly different from zero. Recognizing these limitations, this study is a controlled first step to show the usefulness of training older adults with non-action video games that seem more appropriate for older adults than other genres of video games.

In sum, video game training might be a promising way to improve certain aspects of cognition that decline with age. It will be important for future studies to demonstrate these positive effects with larger sample sizes. Further studies should investigate whether MCI older adults benefited from training with non-action video games as much as healthy older adults. (MCI) benefited from training with video games as much as healthy older adults.

## Author note

The authors would like to inform that they have not had any contact with the package manufacturers of the *Lumosity* cognitive training platform at any time during the duration of the study. This study was conducted independently from them.

### Conflict of interest statement

The authors declare that the research was conducted in the absence of any commercial or financial relationships that could be construed as a potential conflict of interest.
