# Corrigendum: Brain training with non-action video games enhances aspects of cognition in older adults: a randomized controlled trial

**DOI:** 10.3389/fnagi.2015.00082

**Published:** 2015-05-19

**Authors:** Soledad Ballesteros, Antonio Prieto, Julia Mayas, Pilar Toril, Carmen Pita, Laura Ponce de León, José M. Reales, John Waterworth

**Affiliations:** ^1^Studies on Aging and Neurodegenerative Diseases Research Group, Department of Basic Psychology II, Universidad Nacional de Educación a DistanciaMadrid, Spain; ^2^Department of Informatics, Umeå UniversityUmeå, Sweden

**Keywords:** attention, cross-modal oddball attention task, cognitive aging, non-action video games, training support

We would like to clarify that this article as well as the Mayas and colleagues article (Mayas et al., 2014) originated from the randomized controlled intervention study: **clinical trial number:** ClinicalTrials.gov **NCT02007616**. The Mayas et al. (2014) article included the trial number at the end of the Abstract. At the top of this article it said CLINICAL TRIAL ARTICLE but the clinical trial number did not appear due to an involuntary omission, as the trial number was included in the four preliminary versions of the manuscript under revision. We explain that because we would not like to make the reader think that both articles corresponded to results from two different randomized controlled trials. In an intend to avoid misunderstandings, we referred twice in the present publication to the Mayas et al. (2014) paper, the first reference in the Method section and the second in the Discussion. In the Results section, we described briefly the cross-modal oddball attention task and said that, “Results from this task have been reported separately (Mayas et al., 2014)”. Figure 3B corresponding to the results of the cross-modal oddball attention task is similar to Figure 2B of Mayas et al. (2014). In the Brain training article, we included a brief description of the oddball task, a summary of the results and Figure 3B to give the reader a whole picture of the outcomes of our intervention study (that included two speed of processing tasks, the executive control WCST, the cross-modal oddball attention task, several tasks to assess visuospatial working memory – Corsi Blocks, the Jigsaw-puzzle task, and the Rey-Osterrieth Complex Figure Test-, Immediate and Delayed Visual Episodic Memory Tests, and the Wellbeing SPF-IL scale) and referred the reader to Mayas et al. (2014) dedicated specifically to the oddball task for more details.

We would like to explain the differences in the samples showed in the Consort diagrams of the two publications. The reason is that the Mayas et al diagram refers only to the cross-modal oddball task designed to assess alertness and distraction while the Frontier's Consort diagram refers to the whole intervention study with all the outcomes mentioned above. One of the participants of the control group was removed due to the large number of errors in the oddball task, but he was included in the other tasks of the study in which reached inclusion criteria. The same occurred in the trained group. The oddball data were analyzed separately by our coauthors and by the time we sent the results only 15 participants (out of 17) completed the post-evaluation (we could not contact a participant and the other was traveling that week). A few days later, these two participants completed the post-evaluation, but we decided not to include them in the Mayas paper.

## Conflict of interest statement

The authors declare that the research was conducted in the absence of any commercial or financial relationships that could be construed as a potential conflict of interest.
